# Community-based fruit and vegetable prescription programs: a scoping review

**DOI:** 10.1017/jns.2023.81

**Published:** 2023-09-15

**Authors:** Emma Greatorex Brooks, Mark McInerney

**Affiliations:** Department of Clinical Nutrition, Rush University, Chicago, IL, USA

**Keywords:** Community-based, Diet quality, Food insecurity, Fruit and vegetable, Health outcome, Prescription program

## Abstract

Identify and categorise different models of community-based fruit and vegetable prescription programs, to determine variation in terms of methodology, target population characteristics, and outcomes measured. Applying the scoping review methodology, ten electronic databases were utilised to identify community-based fruit and vegetable incentive programs. Results were evaluated by two independent reviewers, using Covidence software. All full-text reviews were completed and documented using the PRISMA-ScR guidelines. Search results were stored and reviewed within the Covidence software. Thirty full-text articles were utilised from the 40 206 identified in the search. Target populations were predominantly female, non-white, and low-income. Considerable heterogeneity was found in both study design and quality. Fruit and vegetable vouchers were utilised in 63 % (*n* 19) of the studies. Prescriptions were primarily provided by community health centres (47 %; *n* 14) or NGOs (307 %; *n* 9) and could be redeemed at farmers’ markets (40 %; *n* 12) or grocery stores (27 %; *n* 8). When measured, diet quality significantly improved in 94 % (*n* 16), health outcomes significantly improved in 83 % (*n* 10), and food security status improved in 82 % (*n* 10) of studies. Providing financial incentives to offset the cost of fresh fruits and vegetables can increase consumption, improve health outcomes, and improve food security status. The majority of studies showed significant improvements in at least one outcome, demonstrating the effectiveness of community-based fruit and vegetable prescription programs. However, the diversity of measurement techniques and heterogeneity of design, dosage, and duration impeded meaningful comparisons. Further well-designed studies are warranted to compare the magnitude of effects among different program methodologies.

## Introduction

It has been well documented that individuals residing in the United States do not consume adequate amounts of fruits and vegetables (F&Vs).^(1–3)^ According to the Dietary Guidelines for Americans (DGAs), adults should consume approximately 1⋅5–2 cup-equivalents of fruits (approximately 120–160 g) and 2–3 cup-equivalents of vegetables (approximately 160–240 g) daily.^(3)^ Presently, 10 % of adults meet the vegetable recommendation and 12⋅3 % of adults meet the fruit recommendation.^(2)^ Adequate intake of F&V may reduce the risk of developing obesity, cardiovascular disease, and diabetes.^(4)^ Schwingshackl *et al.* found that consuming up to 300 g of vegetables per day decreases the risk of mortality by 11 % and consuming up to 250–300 g of fruit per day decreases the risk of mortality by 10 %.^(5)^

Many factors contribute to the inadequate intake of F&V among Americans, however the most prominent barrier cited is cost.^(6)^ It has been shown that a 10 % decrease in price of healthful foods increases consumption by approximately 12 %, and specifically increases consumption of F&V by 14 %.^(7)^ Multiple programs have been created at the state and municipal levels to increase consumption of F&Vs by reducing costs.^(8–19)^ The most widely used program focuses on increasing F&V dollars for Supplemental Nutrition Assistance Program (SNAP) recipients by doubling or matching^(8,9,11–13)^ or providing rebates^(10)^ on dollars spent on F&Vs. These programs often require participants to purchase F&Vs at farmers’ markets.^(8,9,11–13)^ The findings are encouraging as all have shown an increase in F&V purchases and/or consumption among participants.^(8–13)^ Similar results have been seen in programs aimed at increasing consumption of F&Vs among Supplemental Nutrition Program for Women, Infants, and Children (WIC) participants.^(14,15)^ Additionally, programs have been developed to offer direct vouchers of varying amounts^(16–18)^ or rebates^(19)^ to participants. These programs have resulted in an increase of F&V consumption across the United States.^(16–19)^ These data support that decreasing costs may increase purchases and consumption of F&Vs.

The U.S. government has allocated $25 million to pilot additional produce prescription programs which provide F&Vs as well as education.^(20)^ At the present time, there are multiple models, but no clear consensus on which produce prescription programs are the most effective. This in part may be due to the different settings in which programs are administered. Engel and Ruder conducted a scoping review to identify F&V incentive programs for SNAP participants, documenting the different program structures.^(21)^ Whereas Veldheer *et al.* conducted a scoping review of the different types of program healthcare organisations implement to improve F&V access.^(22)^ These reviews provide great insight into two important populations; however, there are still many other produce prescription programs that are not classified into one of these two groups. Therefore, the objective of this scoping review is to identify and categorise the different models of fruit and vegetable prescription programs offered in a community-based setting, in order to understand how they vary in terms of methodology, population characteristics, and outcomes measured.

## Methods

This study utilised the framework for scoping reviews presented by Arksey and O'Malley^(23)^ along with the recommended enhancements from Levac *et al*.^(24)^ The five stages of the framework include: (1) Identifying the research question; (2) Identifying relevant studies; (3) Study selection; (4) Charting the data; and (5) Collating, summarising, and reporting the results.^(23)^ The research objective was defined prior to data collection; however, following Levac *et al.*^(24)^ recommendations, the objective was slightly altered after the relevant studies were identified (stage two), as the original objective was deemed too broad.

### Data collection

Data collection was completed between June 2021 and September 2021. To ensure a robust review, the researchers sought the assistance of the university research librarian. The librarian assisted in developing search streams using text terms and Medical Subject Heading (MeSH) terms. The text terms included: fruit, vegetable, produce, veggie, prescription, health promotion/s, health campaign/s, wellness program/s, incentive*, food voucher/s, food assistance, food stamp/s, school meal/s, school lunch/es, food pantry/ies, food bank/s, and the MeSH terms included: fruit, vegetable, crops, agricultural, health promotion, prescriptions, and food assistance. The researchers used these terms in seven electronic databases including PubMed (Medline), Scopus, Google Scholar, CINAHL, USDA website, and Cochrane Database. Additionally, the Gray Literature Database, OAIster, ProQuest, and MedNar were used to ensure a comprehensive search. In order to capture all possible literature, date parameters were not set for the search.

Due to the nature of a scoping review, the inclusion and exclusion criteria were broad. To be included, the article must describe a community-based program that incentivised F&V consumption, must be written in the English language, and the full-text article must be available. Community-based programs were defined as any F&V prescription being distributed or redeemed within the community setting. During the screening process, F&V prescription programs were defined as an intervention delivering a repeated prescription for F&V to address a diet-related health risk including food insecurity. There were four key elements that determined if a ‘program’ qualified: there must be a prescription for F&V, must require redemption or receipt of F&Vs, there must be a repeated dosage, and participants must have a diet-related health risk including food insecurity. Exclusion criteria included duplicate articles, non-intervention studies, and F&V prescription programs administered exclusively by a healthcare organisation as defined by Veldheer *et al.*^(22)^

All articles from the original search were imported into the Covidence software, which is used to manage systematic reviews.^(25)^ This software removes duplicate articles, maintains accurate count of articles, and allows inclusion and exclusion criteria to be used to categorise articles. Covidence allows for multiple researchers to complete title and abstract screening and full-text review independently of each other by categorising articles as relevant or irrelevant. Additionally, any discordant vote is categorised as a conflict. The initial step of this review consisted of the title and abstract screening, the second step included full-text review, and the final step consisted of data extraction. The two independent researchers met on a weekly basis to discuss all conflicts and reach a consensus on the relevance of the disputed article(s), as well as determine that all necessary data had been extracted.

### Data analysis

There were eleven unique variables collected, most of which were categorical. The variables included duration of intervention (weeks), number of participants, model of F&V prescription program (F&V vouchers, cashback rebates, F&V delivery or collection, or combination), education provided during intervention (none offered, cooking skills/classes, with nutrition professional, handouts, or combination), F&V prescription provider (community health centre, school, federal/state program, NGO or charity, or other), F&V produce provider (food bank/pantry, farmers market, grocery store, or combination), targeted age group of intervention (children, adults, or families), target population's health status (overweight/obese, diabetes, hypertension, multiple health conditions, or no health condition), recipient of government assistance (yes or no), diet quality measured (yes or no), health outcomes measured (yes or no), and food security status measured (yes or no). Extracted data was compiled in a Microsoft Excel spreadsheet. Duration of community-based F&V prescription program and number of participants were reported as means (sd), and frequency distribution was used for the remainder of the variables.

## Results

**Fig. 1.**
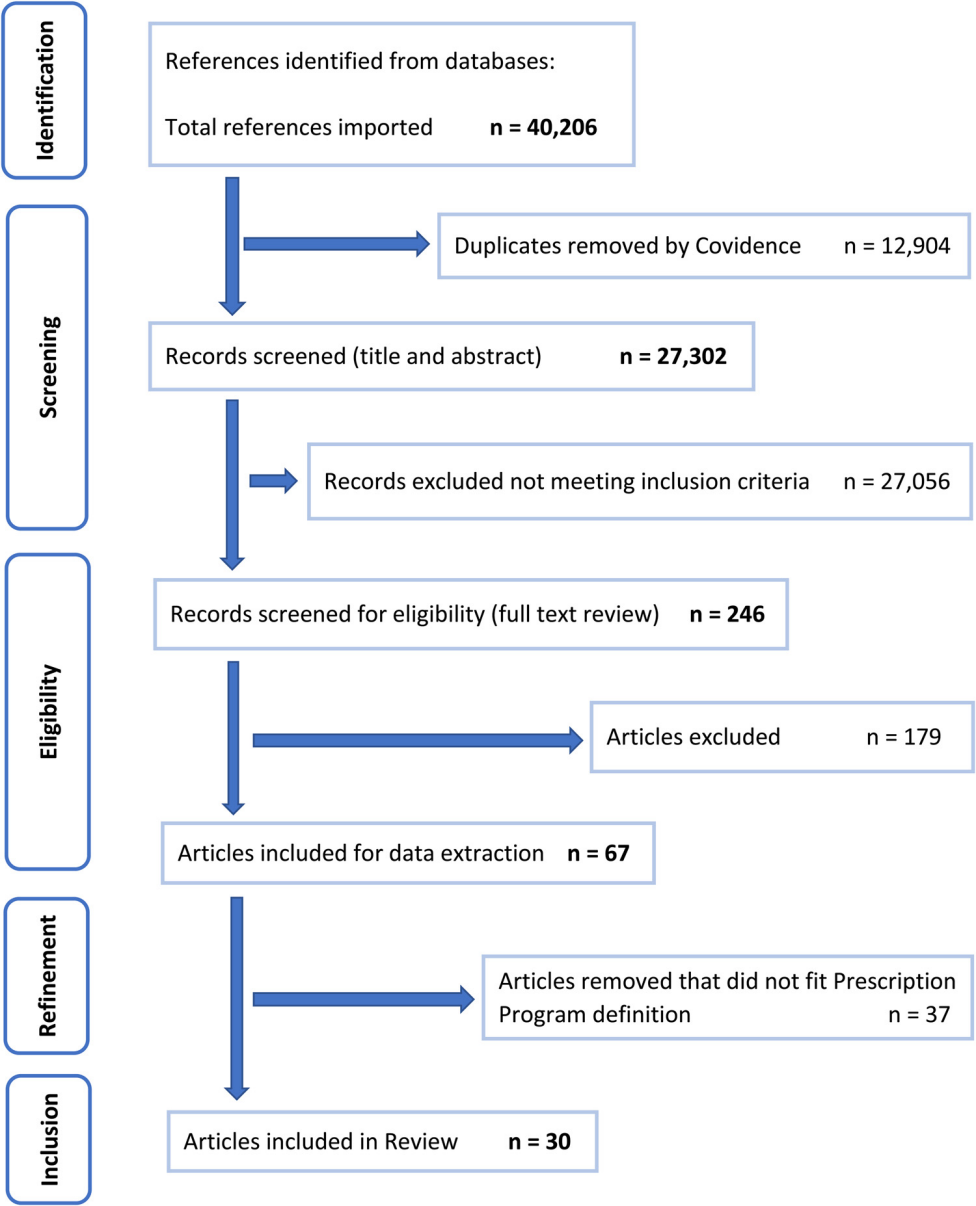
PRISMA flow diagram detailing the study selection process for community-based fruit and vegetable prescription programs (separate document).

The search identified 27 302 unique records after the removal of duplicates by Covidence software, see flow diagram in Fig. 1 detailing the study selection process for community-based F&V prescription programs. A further 27 056 were removed following title and abstract screening against the inclusion criteria, leaving 246 for full-text review which yielded 67 articles for data extraction. During the review, researchers further refined the search parameters to include the definition of ‘prescription program’ as an intervention delivering a repeated prescription for healthy produce to address a diet-related health risk including food insecurity. Following this refinement, thirty articles were included in the final review. A summary of the study characteristics of the community-based F&V prescription programs is presented in Table 1.

**Table 1.** Summary of community-based fruit and vegetable prescription programs

First author (year), country	Study design	Main aim/objective	Sample size	Prescription	Prescription provider	Produce provider	Education provided	Study population description	Outcomes measured	Results
Aiyer^(26)^ (2019), USA	Pre–post mixed methods cohort study	Demonstrate feasibility, acceptability, cost, and impact of food prescription program	242	F&V up to 30 lbs in weight provided for fortnightly collection for 6 months	Community health centre	Food bank /pantry	Nutrition education booklets and recipe cards	Adults >18 years, majority female, Hispanic. Mean household 4⋅6. 24⋅2 % in food assistance programs	FI status Redemption rate	94⋅1 % FI decrease *v.* baseline (*P* < 0⋅01) Average redemption 6⋅5 out of possible 12.
Basu^(27)^ (2021), USA	Pre–post cohort study	Determine if F&V vouchers improve intake and diet quality in two different cities	671	4 × $5 vouchers delivered monthly via mail over a 6-month period	Federal/State funding	Grocery stores and farmers’ markets		Adults >21 years, majority black, female. Median income below FPL	F&V intake	F&V intake +0⋅22 daily cup eq. *v.* baseline (*P* < 0⋅001).
Black^(28)^ (2013), Australia^a^	Retrospective cohort study	Investigate short-term health impacts of F&V subsidy on child health in disadvantaged Aboriginal families	143	Weekly F&V boxes or vouchers, 1–4 children: $40, 5+: $60. Supplied over 12 months	Community health centre	Grocery stores	Cooking/nutrition education sessions led by nutrition professionals	Childre*n* < 17 years in low-income, Aboriginal families	Sick visits Antibiotic use Hb/iron status Well visits BMI	Decrease in sick visits −0⋅6/year (*P* < 0⋅05), Decrease in oral antibiotics −0⋅5 prescriptions/year (*P* < 0⋅05) Increase Hb +3⋅1 g/l (*P* < 0⋅05) No significant change in frequency of preventative visits or BMI.
Black^(29)^ (2013), Australia^a^	Retrospective cohort study	Evaluate if participation in F&V subsidy program improves nutritional biomarkers and dietary behaviour	115	Weekly F&V boxes or vouchers, 1–4 children: $40, 5+: $60. Supplied over 12 months	Community health centre	Grocery stores	Cooking/nutrition education sessions led by nutrition professionals	Childre*n* < 17 years in low-income, Aboriginal families	Nutritional biomarkers F&V intake	Significant increases (*P* < 05) in b-cryptoxanthin (28⋅9 nmol/l, +18 %), vitamin C (10⋅1 mmol/l, +21 %) and lutein–zeaxanthin (39⋅3 nmol/l, +11 %) No significant increases in F&V consumption.
Bowling^(30)^ (2016), USA	Pre–post cohort study	Assess efficacy of healthy food intervention in families enrolled on government assistance program	186	Attendance reward: 40 % rebate on FM spend. Plus $20 bonus for every third visit up to $120 per family	NGO, non-profit, or charity	Farmers’ market	Cooking demos, nutrition education, recipe cards, and handouts	Families enrolled in food assistance program with at least 1 child <12 years	F&V intake Soda intake	Increased vegetable consumption +0⋅28 times/day (*P* = 0⋅005) Reduced soda consumption −0⋅14 times/day (*P* = 0⋅005).
Briefel^(31)^ (2020), USA^b^	Cluster randomised controlled trial	Investigate impact of food box and F&V voucher delivery program on child food insecurity	2859	Monthly food box delivery including $15 F&V voucher over 25 months	Federal/State funding	Grocery stores	Nutrition education handouts	Families with children > 4 years eligible for free school meals	FI status	No significant improvement in child FI *v.* control at 12- or 18-month follow-ups. Adult FI significantly reduced (*P* = 0⋅002) at 12 months (−2⋅8 % *v.* control), not sustained at 18 months.
Bryce^(32)^ (2017), USA	Pre–post cohort study	Examine impact of F&V prescription program on patients with uncontrolled T2D	65	$10 per week for F&V purchases at FM over 13 weeks. Plus $5 participation incentive	Community health centre	Farmers’ market	Cooking demos	Adults >18 years with T2D or HbA1c >6⋅5 %. Majority low-income Hispanic, female participants	HbA1c Weight BP	Decrease in HbA1c concentration 9⋅54 to 8⋅83 % (*P* < 0⋅001) No significant changes in BP and weight.
Bryce^(33)^ (2021), USA	Randomised controlled trial	Assess effectiveness of F&V prescription program on diabetic patients	112	$10 per week to purchase F&V at FM, up to 8 visits over 15 weeks	Community health centre	Farmers’ market	Cooking demos, nutrition education	Adults >18 years with T2D and HbA1c >8⋅0 %. Majority low-income Hispanic, female participants	HbA1c BP, BMI	Decrease in HbA1c concentration 9⋅69 to 9⋅15 % for intervention group (*P* = 0⋅006) No statistically significant changes in BP or BMI for either group.
Burrington^(34)^ (2020), USA	Pre–post cohort pilot study	Examine impact of F&V prescription and education program on rural families	10	$15 weekly online credit for family of 3, $20 for 4, $25 for 5+. F&V delivered for local collection over a 5-month period	School	Community organisation	Cooking/nutrition education sessions led by nutrition professionals	Low-income families with at least one child at risk from chronic disease associated with obesity	F&V intake FI status	Most participants reported consuming more F&Vs Reduction in FI before *v.* after No statistical analysis recorded.
Buscail^(35)^ (2018), France^c^	Randomised controlled trial	Determine whether F&V vouchers modify consumption of F&V in children from low-income households	64	Monthly vouchers (approx. $7 per person) via post to participating families. F2F questionnaires at baseline, 6 months and 1 year	French Ministry of Health	Grocery stores and farmers’ markets	Nutrition education sessions	Families with household income below poverty line	F&V intake	At 1-year child, F&V consumption higher in intervention *v.* control: 4⋅0 (95 % CI 1⋅4, 6⋅0) servings *v.* 2⋅2 (95 % CI 0⋅9, 5⋅0) *P* < 0⋅001. Adults 3⋅0 (95 % CI 0⋅5, 7⋅0) servings *v.* 1⋅9 (95 % CI 0⋅5, 7⋅0) *P* = 0⋅02. Proportion of children defined as low F&V consumers 29⋅4 % (95 % CI 14⋅1, 44⋅7) intervention *v.* control 66⋅7 % (95 % CI 49⋅9, 83⋅5) *P* = 0⋅005.
Buscail^(36)^ (2019), France^c^	Randomised controlled trial	Determine whether F&V vouchers improve food insecurity over a 1-year period	64	Monthly vouchers (approx. $7 per person) via post to participating families. F2F questionnaires at baseline, 6 months and 1 year	French Ministry of Health	Grocery stores and farmers’ markets	Nutrition education sessions	Families with household income below poverty line	FI status	Food security in intervention group significantly improved: baseline FI 85⋅3 % (29) *v.* 61⋅8 % (21) after 1 year *P* = 0⋅03. No difference in the control group.
Cabili^(37)^ (2020), USA^b^	Cluster randomised controlled trial	Investigate whether food box with F&V voucher improves diet quality in children of low-income households	2859	Monthly food box delivery including $15 F&V voucher over 25 months	Federal/State funding	Grocery stores	Nutrition education handouts	Families with children > 4 years eligible for free school meals	F&V intake	F&V (daily cup eq.) and whole grain (oz eq.) intake significantly higher in treatment *v.* control group: F&V combined 2⋅35 *v.* 2⋅25; fruits 1⋅25 *v.* 1⋅20; vegetables 1⋅05 *v.* 1⋅0; whole grains 0⋅73 *v.* 0⋅67 (*P* < 0⋅001).
Cavanagh^(38)^ (2017), USA	Retrospective cohort study	Determine if F&V prescription program is effective in reducing BMI	108	Treatment group received 13 × $7 weekly vouchers for redemption at mobile F&V market	Community health centre	Mobile produce market	Nutrition education sessions led by nutrition professionals	Low-income adults with diagnosis of obesity, hypertension, and/or diabetes	BMI	BMI significantly different pre- *v.* post-intervention in both treatment −0⋅74 kg/m^2^ (2⋅72) and control 0⋅35 kg/m^2^ (1⋅91) groups (*P* < 0⋅0001). Difference between groups also significant *P* = 0⋅02.
Ferdinand^(39)^ (2017), USA	Cross-sectional survey	Assess effectiveness of incentive program on increasing F&V purchasing	96	6 × $4 coupons per week provided to purchase locally grown F&V over a 6-month period	NGO, non-profit, or charity	Farmers’ markets		Adults >18 years receiving SNAP, FMNP, WIC, and/or Medicaid benefits. Majority female, African American participants	F&V purchase F&V intake	63 % of all participants purchased more F&V after the program, 66 % increased variety. SNAP participants increased quantity by 70 % and variety by 63 %. 89 % of participants reported positive changes in consumption of F&V. No pre-intervention consumption data were reported.
Fertig^(40)^ (2021), USA	Pre–post cohort study	Determine the effectiveness of F&V incentive and education program for low-income families	120	3 intervention groups receiving F&V vouchers of $10, $15, or $20 weekly. Participants also enrolled on education program	NGO, non-profit, or charity	Grocery store	Grocery store tour, recipes, and cooking education sessions	Food pantry clients >18 years, majority female participants	F&V intake Attitudes to healthy eating	Pre *v.* post fruit consumption increased by 0⋅79 cups (*P* < 0⋅001). No changes in vegetable consumption. No significant change in attitude towards assembling a healthy meal.
Freedman^(41)^ (2013), USA	Repeated measures cohort study	Evaluate whether financial incentives are effective at increasing F&V consumption among low-income, rural, diabetics	41	$25 F&V vouchers given at baseline (T1) and midpoint (T2) of 22 weeks intervention	Community health centre	Farmers’ market		Adults >18 years with diabetes, majority female, African American participants from low-income households	F&V intake	Marginally significant increase T1 to T2 in mean F&V servings; 5⋅9 to 7⋅5 (*P* = 0⋅07). Participants categorised as increasers (≥0⋅5 servings/day at T2/3 *v.* T1) and non-increasers. Odds of being an increaser higher for those using only vouchers as payment (OR 38⋅8, 95 % CI 3⋅35, 445⋅0) and visiting FM more often (OR 2⋅07, 95 % CI 1⋅09, 3⋅95).
Jones^(42)^ (2020), USA	Pre–post cohort study	Investigate the impact of paediatric F&V prescription program	122	Families given weekly F&V vouchers worth $1 per person per day (up to max $5 per day) over a 6-month period	Community health centres	Grocery stores and farmers’ markets	Nutrition education sessions	Childre*n* ≤ 6 years in Navajo Nation with low or very low FS	F&V intake FI status BMI	Mean (sd) F&V consumption increased from 5⋅2 (2⋅1) to 6⋅8 (2⋅2) servings/day (*P* < 0⋅001). Household FI decreased from 82 to 65 % (*P* < 0⋅001). Of children classified as overweight or obese at baseline (*n* 58), 38 % achieved healthy BMI *z*-score at end (*P* < 0⋅05), their mean BMI percentile decreasing from 95⋅6 (4⋅1) to 73⋅1 (28⋅7), (*P* < 0⋅001).
Kerr^(43)^ (2020), USA	Prospective cohort study	Investigate whether F&V prescription improves cardiometabolic outcomes in adults with or at risk of T2D	47	10 × weekly F&V prescriptions enough for 21 servings non-starchy veg for collection from community site	Community health centres	Wholesale direct from farms		Adults with or at risk of T2D	F&V intake WC BP Weight HbA1c Glycaemic control FI	Proportion consuming ≥1 serving/day of vegetables increased 15 to 50 % pre *v.* post (*P* < 0⋅0001). WC decreased −0⋅77 cm (95 % CI −1⋅42, 0⋅12, *P* = 0⋅022). SBP decreased −2⋅42 mm Hg (95 % CI −4⋅56, 0⋅28, *P* = 0⋅037). Weight decreased −0⋅4 kg (−0⋅7 to –0⋅04, *P* = 0⋅029) in women. In participants with HbA1c > 7⋅0 %: HbA1c decreased −0⋅35 % (−0⋅8 to –0⋅1, *P* = 0⋅009) Participants with CGM data (*n* 40), time in range 70–180 mg/dl improved (97⋅4 to 98⋅9 %, *P* < 0⋅01) Participants with low or very low FS fell from 35 to 13 % (*P* < 0⋅001).
LaBarba^(44)^ (2019), USA	Pre–post cohort study	Determine impact of F&V prescription program on dietary quality in low-income expectant mothers	25	Monthly $40 F&V vouchers on attending obstetric appointment, from first trimester to 6 weeks postpartum	Community health centres	Grocery store	Grocery store tour, nutrition education sessions	Low-income, pregnant women. Majority Hispanic participants	F&V intake	F&V intake increased baseline *v.* T1 (pre-delivery); fruit 2⋅0 to 2⋅61 daily servings (*P* < 0⋅05); vegetable 1⋅44 to 2⋅39 (*P* < 0⋅001). Baseline *v.* T2 (6 weeks postpartum); fruit increased 2⋅0 to 2⋅5 daily servings (*P* < 0⋅05) vegetable 1⋅44 to 2⋅28 (*P* < 0⋅01).
Orsega-Smith^(45)^ (2020), USA	Pre–post cohort study	Evaluate F&V prescription program offered via paediatrician to low-income families	41	Monthly F&V boxes collected from doctors’ office containing 15–25 lb F&V per box	Community health centres	Food bank /pantry	Cooking and nutrition education sessions	Low-income families with ≥2 children or overweight adult. Majority white participants	F&V intake FI status	Increases in F&V intake (servings/day) pre *v.* post: Adult vegetables 2⋅22 ± 1⋅24 to 2⋅44 ± 1⋅03 (*P* < 0⋅001), fruit 2⋅05 ± 0⋅97 to 2⋅46 ± 0⋅92 (*P* < 0⋅05). Children; fruit 2⋅51 ± 1⋅21 to 2⋅77 ± 1⋅16 (*P* < 0⋅05). No significant difference in vegetable consumption. Perceptions of FI improved
Palar^(46)^ (2017), USA	Prospective cohort study	Assess impact of food intervention program to improve nutrition, mental health, and health behaviours	52	Healthy meals and snacks fulfilling 100 % daily caloric requirements for collection 2× per week over a 6-month period.	NGO, non-profit, or charity	Food bank/pantry		Adults >18 years with HIV and/or T2D, majority male participants	Diet quality FI status ART adherence	Frequency of consumption of fats decreased (*P* = 0⋅003), frequency consumption F&V increased (*P* = 0⋅011). Among people with diabetes, frequency of sugar consumption decreased (*P* = 0⋅006) VLFS decreased 59⋅6 to 11⋅5 % (*P* < 0⋅0001) ART adherence ≥95 % increased 46⋅7 to 70⋅0 % (*P* = 0⋅046).
Richie^(47)^ (2019), USA	Pre-experimental	Measure impact of FM voucher program on health outcomes in low-income population	308	F&V vouchers redeemable at FM $1 per day per person in HH, or $2 for study participation. Over a 6-month period	Community health centres	Farmers’ market		Low-income adults >18 years with diagnosis of Diabetes, hypertension and/or obesity	FBG WC, BMI, weight, BP	Participants’ mean (sd) FBG decreased pre *v.* post −8⋅92 (5⋅36) mg/dl (*P* = 0⋅023) WC decreased −0⋅26 (0⋅17) inches (*P* = 0⋅001). No significant changes in BP, BMI, or weight.
Ridberg^(48)^ (2018), USA	Pre–post retrospective cohort	Assess food security status before and after paediatric F&V prescription program	578	Families received $0⋅50 to $1⋅00 vouchers per person per day redeemable at local FM over 4–6 months	NGO, non-profit, or charity	Farmers’ market	Nutrition education sessions	Low-income families with children aged 2–18 years, at least one child to be classified as obese or overweight	FI status	Mean FS score increased 0⋅72–0⋅81 (*P* < 0⋅001). High or marginal FS status increased 58–76 % (*P* < 0⋅001), low FS decreased by 33–22 % (*P* < 0⋅001), and very low FS decreased by 9–1 % (*P* < 0⋅001). Families attending 5–6 visits had +0⋅7 FS change score *v.* those attending 1–2 visits (95 % CI 0⋅01, 0⋅14, *P* < 0⋅05).
Ridberg^(49)^ (2019), USA	Pre–post retrospective cohort	Assess F&V intake before and after paediatric F&V prescription program	883	Families received $0⋅50 to $1⋅00 vouchers per person per day to be redeemed at local FM over 4–6 months	NGO, non-profit, or charity	Farmers’ market	Nutrition education sessions	Low-income families with children aged 2–18 years, at least one child to be classified as obese or overweight	F&V intake	Mean (sd) fruit intake pre *v.* post; 1⋅6–1⋅7 cups (0⋅13 (1⋅2), 95 % CI 0⋅05, 0⋅21, *P* < 0⋅001); vegetable intake 1⋅2–1⋅3 cups (0⋅13 (1⋅1) 95 % CI 0⋅06, 0⋅21, *P* < 0⋅001). Combined mean F&V intake increased 0⋅26 cups pre *v.* post (95 % CI 0⋅13, 0⋅39, *P* < 0⋅001). Pre *v.* post meeting recommendations; 78 % *v.* 86 %. +0⋅32 cups for each additional clinic visit (95 % CI 0⋅20, 0⋅45; *P* < 0⋅001).
Saxe-Custack^(50)^ (2019), USA	Pre–post longitudinal cohort	Determine impact of F&V prescription program on fruit consumption in children	114	$15 F&V voucher prescribed at paediatrician visits, to be redeemed at FM	Community health centre	Farmers’ market		Children aged 7–18 years	F&V intake FI status	Mean (sd) daily servings whole fruit increased baseline *v.* 6 months 0⋅62 (0⋅69), to 0⋅81 (0⋅64), *P* = 0⋅029. No statistically significant changes in total fruit, fruit juice, or vegetable consumption No significant change in FI status.
Slagel^(51)^ (2021), USA	Non-randomised control trial	Assess impact of pilot F&V prescription program on low-income adults with chronic health conditions	24	Monthly F&V vouchers of $1 per day per household member for 6 months	NGO, non-profit, or charity	Farmers’ market	Cooking and nutrition education sessions	Adults with overweight/obesity, diabetes, prediabetes, hypertension, and/or hyperlipidaemia	F&V intake	Mean (sd) Pre–post F&V intake higher in Intervention *v.* control; 0⋅81 (0⋅91) servings/day *v.* −0⋅25 (0⋅99) *P* = 0⋅02. Increases intervention group pre *v.* post total vegetable intake, dark green vegetable intake, and HEI score (*P* = 0⋅005). Increased knowledge of F&V preparation (*P* = 0⋅02), increase in F&V purchases (*P* = 0⋅05).
Snailer^(52)^ (2019), USA	Pre–post cohort study	Determine whether F&V prescription program lowers HBA1c levels in food insecure participants with T2D	14	Monthly F&V vouchers worth $1 per day per HH member for redemption at FM over 6-month program	Community health centre	Farmers’ market	Cooking and nutrition education sessions	Food insecure adults with T2D and HbA1c > 7⋅0 %. Majority white, female participants	HbA1c	Average decrease in HbA1c 1⋅85 % (95 % CI −2⋅69, −1⋅01) *P* = 0⋅0004. Linear model showed a $10 increase in average monthly redemptions associated with 1⋅4 % (95 % CI 0⋅5, 2⋅4) decrease in HbA1c *P* = 0⋅006.
Trapl^(53)^ (2018), USA	Pre–post cohort study	Evaluate F&V redemption and consumption among food insecure adults with HTN	224	Weekly $10 F&V vouchers for 3 months; up to $120, redeemable at local FM	Federal/State funding	Farmers’ market	Nutrition education sessions	Food insecure and hypertensive adults. Majority black, female participants	F&V intake	Mean servings (sd) fruit increased pre *v.* post 1⋅6 (1⋅3) to 2⋅4 (1⋅2), *P* < 0⋅001. Vegetables increased 1⋅7 (1⋅1) to 2⋅5 (1⋅3), *P* < 0⋅001. Fast-food servings decreased 1⋅3 (1⋅4) to 0⋅7 (1⋅0), *P* < 0⋅001.
Xie^(54)^ (2021), USA	Prospective cohort study	Assess produce prescription program utilisation, healthy food purchases, and health outcomes	699	$40 monthly added to SNAP EBT card or store card for WIC approved F&V over a 1-year period	NGO, non-profit, or charity	Grocery stores		SNAP eligible adults aged ≥18 years	F&V intake (purchasing data as proxy) HbA1c, BMI, BP	Frequent spenders *v.* sometimes spenders: higher F&V spending (B=$8⋅77, *P* < 0⋅001), higher F&V expenditure share (B = 3⋅3 %, *P* = 0⋅007), and more unique F&V purchases (B = 2⋅52, *P* < 0⋅001). No significant relationships between program utilisation and health outcomes.
York^(55)^ (2021), USA	Prospective cohort study	Assess feasibility and impact of farming for life pilot program among Latino adults with T2D	21	Weekly F&V box, value $31 for collection by participants over a 12-week period.	NGO, non-profit, or charity	Direct from local organic farms		Latino adults with T2D, majority female participants	BP FI status HbA1c, BMI, WC	Reduction in systolic BP (*P* = 0⋅03) and diastolic BP (*P* = 0⋅01) FI improved in 57 % of participants. No statistically significant changes in weight, WC or HbA1c.

F&V, fruit and vegetables; FI, food insecurity; FPL, federal poverty level; Hb, haemoglobin; BMI, body mass index; FM, farmers’ market; NGO, non-governmental organisation; T2D, type 2 diabetes mellitus; HbA1c, glycated haemoglobin; BP, blood pressure; F2F, face to face; SNAP, Supplemental Nutrition Assistance Program; FMNP, farmers’ market nutrition program; WIC, women, infants, children special supplemental nutrition program; FS, food security; WC, waist circumference; CGM, continuous glucose monitor; HIV, human immunodeficiency virus; VLFS, very low food security; ART, antiretroviral therapy; HH, households; FBG, fasting blood glucose; HTN, hypertension; EBT, electronic benefits transfer.

^abc^Superscript letters denote studies carried out on the same intervention.

The thirty studies included in this review comprise twenty-seven unique interventions taking place in the USA (*n* 25), France (*n* 1), and Australia (*n* 1). The mean (sd) F&V program duration was 35⋅2 (25⋅0) weeks, with the most common length being 26 weeks. The number of study participants ranged from 10^(34)^ to 2,859,^(31,37)^ with a mean (sd) number of participants of 366 (713). Table 2 shows the frequency distribution of categorical variables within program methodology, population characteristics, and outcomes measured in the community-based F&V prescription programs.

**Table 2.** Distribution of variables within program methodology, population characteristics and outcomes measured in community-based F&V prescription programs

Variable description	Frequency	
	(%)	(*n*)
Model		
F&V Vouchers	63	(19)
Delivery and/or collection	27	(8)
Cashback rebate	7	(2)
Combination	3	(1)
Education		
Session with nutrition professional	10	(3)
Handouts or leaflets	10	(3)
Cooking skills or classes	3	(1)
Combination	47	(14)
None offered	30	(9)
Prescription provider		
Community health centre	47	(14)
NGO/charity	30	(9)
Federal/State program	13	(4)
School	3	(1)
Other	7	(2)
Produce provider		
Farmers’ market	40	(12)
Grocery store	27	(8)
Food bank/pantry	10	(3)
Combination/other	23	(7)
Target population age		
Children	13	(4)
Adults	57	(17)
Families	30	(9)
Target population health status		
Diabetes	23	(7)
Overweight or obesity	10	(3)
Hypertension	3	(1)
Multiple health conditions	13	(4)
Not a selection criterion	50	(15)
Dietary quality outcome		
Significant improvement	53	(16)
No improvement	3	(1)
Not measured	43	(13)
Health outcome		
Significant improvement	33	(10)
No improvement	3	(1)
Not measured	63	(19)
Food security outcome		
Significant improvement	33	(10)
No improvement	3	(1)
Not measured	63	(19)

### Program methodology

The most common F&V prescription intervention model was F&V vouchers (*n* 19), which provide vouchers to participants to be redeemed for fresh fruit and vegetables.^(27,31–33,35–42,44,47,50–54)^ The second most frequent model was delivery and/or collection (*n* 8), with fresh F&V delivered to participants or made available for collection.^(26,28,29,34,43,45,46,55)^ Lastly, two studies used a cashback rebate on F&V purchases, via EBT card and associated with enhancement to SNAP benefits.^(48,49)^ In addition, one study employed a combination of these models.^(30)^

Another variable within program methodology is provision of education, ranging from simple distribution of information, cooking tips and recipes via handouts^(26,31,37)^ (*n* 3), to cooking classes^(32)^ (*n* 1), to regularly scheduled education sessions led by nutrition professionals^(38,48,49)^ (*n* 3). The most common approach (*n* 14) was to employ a combination of formats.^(28–30,33–36,40,42,44,45,51–53)^

Providers of both prescriptions and produce varied depending on the program. The most common provider of F&V prescriptions (*n* 14) was community health centres,^(26,28,29,32,33,38,41–47,50,52)^ followed by non-governmental organisations, other non-profit, and charitable foundations^(30,39,40,46,48,49,51,54,55)^ (*n* 9). Other providers included federal/state programs^(27,31,37,53)^ (*n* 4) and schools^(34)^ (*n* 1). The produce mainly came from one of three sources: Farmers’ markets^(30,32,33,39,41,47–53)^ (*n* 12), grocery stores^(27–29,31,37,40,44,54)^ (*n* 8), or food banks and pantries^(26,45,46)^ (*n* 3), with several (*n* 7) utilising a combination.^(34–36,38,42,43,55)^

### Population characteristics

The majority of F&V prescription interventions exclusively included adults^(26,27,32,33,38–41,43,44,46,47,51–55)^ (*n* 17), 30 % targeted families^(30,31,34–37,45,48,49)^ (*n* 9), and children made up the target population for the smallest proportion of the studies^(28,29,42,50)^ (*n* 4). Exactly half of the studies targeted populations with a specific health status, most commonly type 2 diabetes^(32,33,41,43,52,54,55)^ (*n* 7), followed by overweight and obesity^(34,48,49)^ (*n* 3), others included hypertension^(53)^ and HIV-positive status.^(46)^ Participation in a government assistance program was required in 27 % (*n* 8) of studies,^(28–31,34,37,39,54)^ although all of the studies described one or more indicators of low income or food insecurity measurement.

Other trends were seen within the target populations; however, these could not be collated as frequencies due to the differences in reporting between studies. Target populations were majority female,^(26,27,30,32,33,35,36,39,40–44,47–55)^ and majority non-white,^(26–30,32,33,38,39,41–44,48,49,50,53–55)^ where specified.

### Study designs

There was considerable heterogeneity in the design of the different studies, with three main study designs used. The first being non-randomised observational study designs (*n* 24);^(26–30,32,34,38,39–50,52–55)^ the second, randomised controlled trials (*n* 5);^(31,33,35–37)^ and the third, non-randomised clinical trial (*n* 1).^(51)^

### Outcomes measured

Change in dietary quality was measured in the majority of studies^(27,29,30,34,35,37,40–46,49,50,51,53)^ (*n* 17), only one of which reported no statistically significant improvement in diet quality.^(34)^ The number and type of instruments used to measure diet quality varied widely, as did their levels of reliability, these included food frequency questionnaire (FFQ)^(35)^ (*n* 1), validated 24-hour recall methods^(27,29,40,51)^ (*n* 4), HEI scores^(27,51)^ (*n* 2), and nutritional biomarkers^(29)^ (*n* 1). Several of the studies used externally validated screeners to measure F&V intake, including NCI dietary screener^(37,41,49)^ (*n* 3), the Block screener^(50)^ (*n* 1), and the Behavioural Risk Factor Surveillance System (BRFSS)^(42)^ (*n* 1), in addition to other instruments not often cited in the literature^(44,46,53)^ (*n* 3). Internally designed, non-validated surveys were used to assess F&V intake in the remainder^(30,34,43,45)^ (*n* 4).

Outcomes related to health status were also measured in eleven of the studies,^(28,32,33,38,42,43,46,47,52,54,55)^ with all but one^(54)^ reporting a statistically significant improvement in at least one health measure. All outcome measures were conducted by a trained medical or research staff, or retrieved from medical records, none relied on self-report. The different health outcome measures included frequency of sick visits to healthcare provider and/or hospital attendances^(28)^ (*n* 1), number of oral antibiotic prescriptions^(28)^ (*n* 1), iron status^(28)^ (*n* 1), weight^(32,43,47,55)^ (*n* 4), BMI^(28,38,42,46,47,54,55)^ (*n* 7), blood pressure^(32,33,43,47,54,55)^ (*n* 6), glycated haemoglobin (HbA1c) level^(32,33,43,46,52,54,55)^ (*n* 7), waist circumference^(43,47,55)^ (*n* 3), glycaemic control (continuous glucose monitor and fasting blood sugar measurements)^(43,46,47)^ (*n* 3), and antiretroviral therapy adherence^(46)^ (*n* 1).

Food security status was also an outcome of interest in eleven of the studies,^(26,31,34,36,42,43,45,46,48,50,55)^ with only one study not reporting a statistically significant improvement in food security status.^(50)^ There was less variation in the measurement instruments compared to diet quality measurements, with the majority using the widely used USDA 18-item Household Food Security Survey^(31,36,46,48,55)^ (*n* 5). Others used the 2-item^(26)^ (*n* 1) and 6-item^(42,43,50)^ (*n* 3) versions which have been shown to have high sensitivity and specificity when compared to the original Household Food Security Survey tool.^(56,57)^ Two studies used internally designed, non-validated surveys.^(34,45)^

## Discussion

The results of this review show encouraging outcomes for individuals who receive and utilise produce prescriptions from community-based organisations. Similar to previous studies that showed increased consumption of F&Vs when financial incentives are provided,^(7)^ SNAP benefits are increased,^(8–13)^ WIC benefits are increased,^(14,15)^ or when direct vouchers^(16–18)^ or rebates^(19)^ are provided, the majority of community-based produce prescription programs showed an increase in F&V consumption when a financial incentive was offered.^(27,34,35,37,41,42,44–46,49,51,53)^ A minority of the studies only showed increases in vegetable^(30,43)^ or fruit consumption,^(40,50)^ with even fewer showing no statistically significant increases in overall F&V intake.^(29,40)^ Also, two studies showed an increase in F&Vs purchased but did not report pre–post consumption data.^(39,54)^ These results further support the benefits of providing financial assistance to offset the often-cited barrier of purchasing F&V, which is cost.^(6)^ The community-based produce prescription programs are in fact assisting individuals increase F&V consumption moving them closer to meeting the DGA's recommendations.^(3)^

Lessening the financial burden associated with purchasing groceries may directly lead households or individuals towards improved food security status. This trend was consistently seen in ten of the articles reviewed,^(26,34,36,37,42,43,45,46,48,55)^ especially among individuals classified as very low food secure and low food secure.^(43,46,48)^ Interestingly, the article that did not find statistically significant improvement in food security status also did not see an increase in F&V consumption.^(50)^ Whereas, all other studies that measured both food security status and F&V intake showed statistically significant improvements in both indices.^(34,42,43,45,46)^ These results indicate a possible correlation between the two variables.

As previously discussed, increasing F&V intake may reduce the risk of developing certain chronic diseases and decrease overall mortality risk.^(4,5)^ Although many of the studies reviewed measured certain health-related indices,^(28,29,32,33,38,42,43,46,47,52,54,55)^ only four measured health indicators and actual F&V intake.^(42,43,46,54)^ Additionally, only Jones *et al.*^(42)^ (BMI *z*-score) and Kerr *et al.*^(43)^ (waist circumference, systolic blood pressure, HbA1c, glucose, and weight in women) showed statistically significant improvements in health outcomes with an increase in F&V consumption. Diabetes management was a focus for many of the studies,^(32,33,43,47,52,55)^ with five showing improvement in HbA1c^(32,33,43,52)^ and fasting blood glucose levels^(47)^ during the produce prescription intervention timeframe. Though encouraging, it is difficult to determine what specific factors contributed to improved glycaemic control during these interventions, as dietary data was not reported.

There are clearly opportunities to utilise community-based incentive programs to improve both dietary quality, food security status, and health outcomes, which has been supported by previous scoping reviews exploring the application of F&V prescription programs in other areas.^(21,22)^ However, they too have made similar comments regarding the variety and heterogeneity in design and quality of programs. Focusing specifically on healthcare organisations, Veldheer *et al.*^(22)^ found most studies assessing dietary quality showed improvements, although health-related outcomes were more mixed. Engel and Ruder found some measure of positive impact in all but one of the studies in their review of F&V incentive programs for SNAP participants.^(21)^ The current review adds to the literature that providing produce prescriptions in a variety of community settings, outside of healthcare organisations and SNAP, improves the overall intake of F&V, food security status, and certain health outcomes. The similarities among the programs could provide a standardised produce prescription blueprint to be implemented across different settings.

This scoping review has some definite strengths, it was conducted using the methodological framework from Arksey and O'Malley^(23)^ with enhancements from Levac *et al.*^(24)^, and was conducted following the PRISMA-ScR guidelines and checklist.^(58)^ The comprehensive database search was guided by a specialist, and all screening, data extraction, and mapping were conducted by two reviewers independently. There are some limitations, the search revealed several abstracts that met the criteria but were unable to locate the full-text article and therefore not included, this may have meant some relevant data could have been missed. In addition, following the broad format for a scoping review may have meant that search was too wide, a more targeted research question may have helped reduce the heterogeneity found across the interventions. Finally, no meaningful statistical analysis could be completed due to the lack of experimental design of most of the studies, plus the limited comparison groups in the non-experimental study designs.

## Conclusion

This scoping review of community-based interventions delivering a repeated prescription for healthy produce to address a diet-related health risk found that most studies showed a significant improvement in one or more of the outcomes measured. However, the diversity of measurement tools made meaningful comparisons of the effectiveness of the different programs impossible. In addition, the heterogeneity of program design, dosage and duration, and the format and frequency of education, also make meaningful comparisons difficult. So, while most outcomes showed statistically significant improvements, these limitations raise questions as to the strength of the evidence.

In summary, further study is warranted to compare the magnitude of effects with the different program methodologies, and the education component requires further investigation to understand its contribution to program effectiveness. Most of the studies included in this review were non-randomised and did not have a control group, more rigorously designed and adequately powered RCTs are required to accurately evaluate these types of intervention. Additionally, there are other barriers to the intake of fresh fruit and vegetables besides cost, including access and acceptability, these also require further research. After completion of this review, recommendations for next steps would be to conduct larger randomised control trials to determine the effectiveness of produce prescription programs.
